# Foodprint 2.0: A computational simulation model that estimates the agricultural resource requirements of diet patterns

**DOI:** 10.1371/journal.pone.0306097

**Published:** 2024-09-04

**Authors:** Zach Conrad, Songze Wu, LuAnn K. Johnson, Julia F. Kun, Eric D. Roy, Jessica A. Gephart, Nayla Bezares, Troy Wiipongwii, Nicole Tichenor Blackstone, David C. Love

**Affiliations:** 1 Department of Kinesiology, William & Mary, Williamsburg, VA, United States of America; 2 Global Research Institute, William & Mary, Williamsburg, VA, United States of America; 3 Independent Contractor, Warren, Minnesota, United States of America; 4 College of Arts & Sciences, William & Mary, Williamsburg, VA, United States of America; 5 Gund Institute for Environment, University of Vermont, Burlington, VT, United States of America; 6 Department of Civil & Environmental Engineering, University of Vermont, Burlington, VT, United States of America; 7 Rubenstein School of Environment & Natural Resources, University of Vermont, Burlington, VT, United States of America; 8 Department of Environmental Science, American University, Washington, DC, United States of America; 9 School of Aquatic and Fishery Sciences, University of Washington, Seattle, WA, United States of America; 10 Friedman School of Nutrition Science and Policy, Tufts University, Boston, MA, United States of America; 11 Department of Environmental Science and Engineering, Johns Hopkins Bloomberg School of Public Health, Baltimore, MD, United States of America; 12 Center for a Livable Future, Johns Hopkins Bloomberg School of Public Health, Baltimore, MD, United States of America; Sichuan University, CHINA

## Abstract

Reducing the environmental pressures stemming from food production is central to meeting global sustainability targets. Shifting diets represents one lever for improving food system sustainability, and identifying sustainable diet opportunities requires computational models to represent complex systems and allow users to evaluate counterfactual scenarios. Despite an increase in the number of food system sustainability models, there remains a lack of transparency of data inputs and mathematical formulas to facilitate replication by researchers and application by diverse stakeholders. Further, many models lack the ability to model multiple geographic scales. The present study introduces Foodprint 2.0, which fills both gaps. Foodprint 2.0 is an updated biophysical simulation model that estimates the agricultural resource requirements of diet patterns and can be adapted to suit a variety of research purposes. The objectives of this study are to: 1) describe the new features of Foodprint 2.0, and 2) demonstrate model performance by estimating the agricultural resource requirements of food demand in the United States (US) using nationally representative dietary data from the National Health and Nutrition Examination Survey from 2009–2018. New features of the model include embedded functions to integrate individual-level dietary data that allow for variance estimation; new data and calculations to account for the resource requirements of food trade and farmed aquatic food; updated user interface; expanded output data for over 200 foods that include the use of fertilizer nutrients, pesticides, and irrigation water; supplementary files that include input data for all parameters on an annual basis from 1999–2018; sample programming code; and step-by-step instructions for users. This study demonstrates that animal-sourced foods consumed in the US accounted for the greatest share of total land use, fertilizer nutrient use, pesticide use, and irrigation water use, followed by grains, fruits, and vegetables. Greater adherence to the Dietary Guidelines for Americans was associated with lower use of land and fertilizer nutrients, and greater use of pesticides and irrigation water. Foodprint 2.0 is a highly modifiable model that can be a useful resource for informing sustainable diet policy discussions.

## Introduction

Agriculture accounts for over 40% of global land use and nearly two-thirds of freshwater withdrawals, [1] and concerns about the environmental challenges posed by food systems have risen to the forefront of policy discussions across the globe. The United Nations Sustainable Development Goals for 2030 call for improved agricultural stewardship and management of natural resources along with improved nutritional outcomes [2]. In the United States (US), food demand drives over one quarter of land use and freshwater withdrawals, [3] and similarly broad policy actions have begun to emerge. For example, the Biden-Harris administration released the National Strategy on Hunger, Nutrition, and Health in September 2022, which calls for more research to evaluate nutrition-environment interactions [4]. The US Departments of Agriculture (USDA) and Health and Human Services (US HHS) have also initiated several programs to address this need [5, 6].

These policy discussions are informed by analytic frameworks that evaluate the relationships between diet patterns and environmental outcomes [7–10]. These can take different forms but computational models are among the most common, [8] which use data and mathematics to represent complex systems and allow users to perform simulations by modifying one or several variables at a time [11]. Although the number of food system sustainability models has increased recently, there remain several gaps that need to be filled to optimally inform policy discussions. These include greater transparency of data inputs and mathematical formulas to facilitate replication by researchers and application by diverse stakeholders [7, 10] and improved capacity to model multiple geographic scales (e.g., community, national, and global) [8, 10].

The Foodprint model [12] addresses both of these gaps. Foodprint is a biophysical food system model developed by Peters et al. [12] that estimates the agricultural land needed to feed a given population a given diet pattern. The model accepts user-inputted dietary data on a per-capita basis, and then embedded calculations transform the associated foods as they move backwards through the US food system from consumer foods to processed products to agricultural commodities, and ultimately to the land required to produce the associated crops. Since its inception others have used Foodprint for a variety of research purposes, such as evaluating the agricultural capacity for local food systems, [13] the agricultural resource requirements of food loss and waste, [14] and the relationship between diet quality and agricultural resource use.[15]

Despite the versatility of Foodprint’s original design, new features are needed to improve its robustness and usability, which we introduce in the present study. These include embedded functions to integrate individual-level dietary data, which also allow for variance estimation; new data and calculations to account for the land requirements of food trade and farmed aquatic foods; updated user interface; expanded output data that include the use of fertilizer nutrients, pesticides, and irrigation water associated with US food demand; output files for over 200 foods as well as summary output charts for each food category; supplementary files that include raw and summary data for each parameter on an annual basis from 1999–2018; and step-by-step instructions for users with different data needs. This new version of the model is called Foodprint 2.0.

The objectives of this study are to 1) describe the new features of Foodprint 2.0, and 2) demonstrate model performance by estimating the use of agricultural land, fertilizer nutrients, pesticides, and irrigation water associated with food demand in the US using ten years of nationally representative dietary data from the National Health and Nutrition Examination Survey (NHANES, 2009–2018).

## Methods

### Model structure

**Fig 1:**
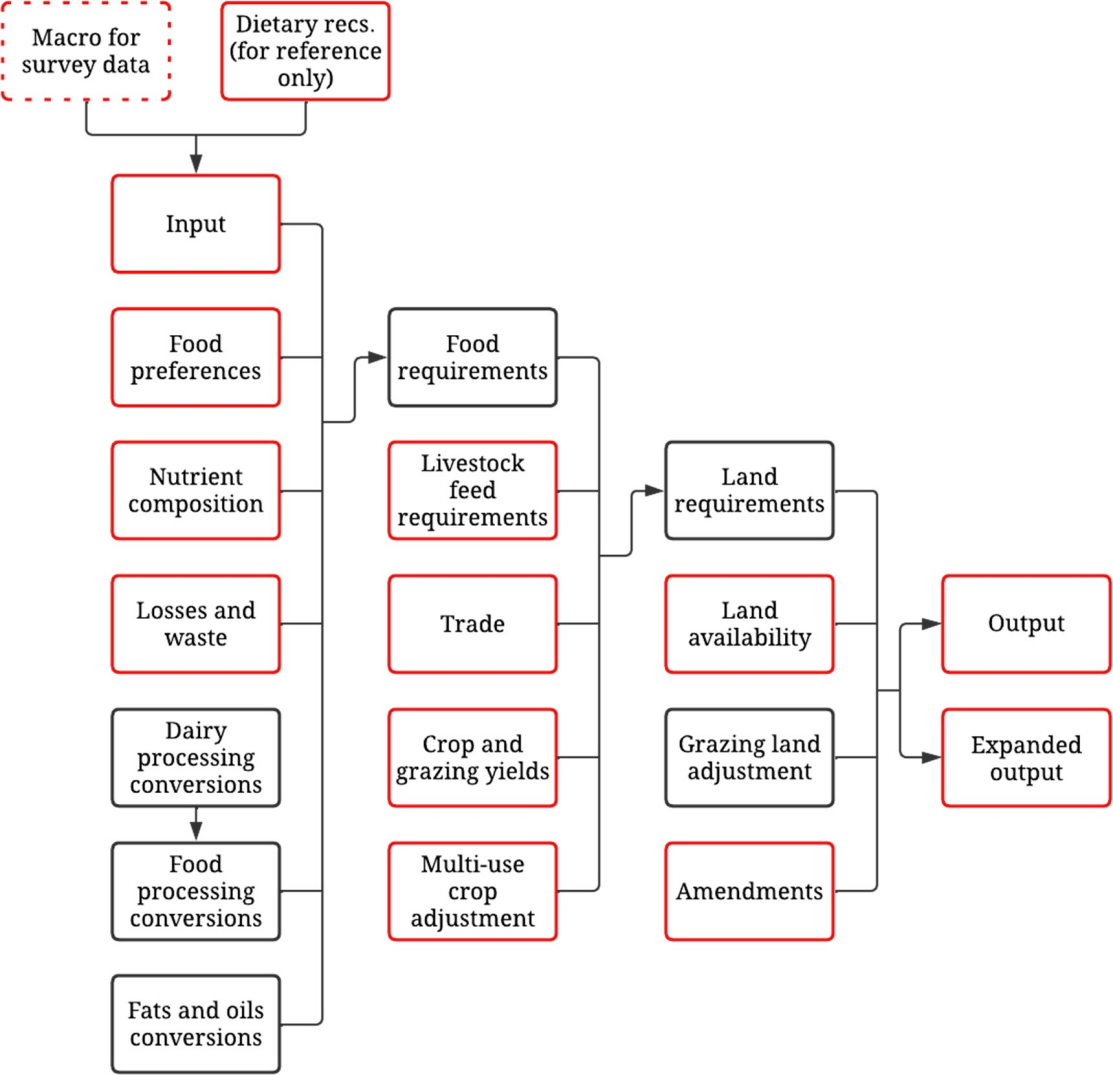
Structure of Foodprint 2.0. Each box represents a distinct worksheet in the model. Those with a dashed border represent an embedded macro and those with a red border represent worksheets with new features or updated data. Foodprint 2.0 and supporting documents can be accessed at: https://doi.org/10.17026/dans-zmh-tzn3.

Foodprint 2.0 is a spreadsheet model that estimates the amount of agricultural land, fertilizer nutrients (nitrogen, phosphorus-P_2_O_5_, potash-K_2_O, and sulfur), pesticides (sum of herbicides, insecticides, and fungicides), and irrigation water needed to meet user-defined diet patterns in the US. These output data are provided for each of the 208 foods and 10 food categories included in the model. The model contains 20 integrated worksheets that use embedded data and calculations (**Fig 1**). Users enter dietary data into the *Input* worksheet and the embedded computations in subsequent worksheets transform the mass quantity of these foods in stepwise fashion as they move backwards through the food system from being consumer foods to processed products to agricultural commodities, and ultimately to the agricultural resources needed to produce these commodities. Each of these worksheets is described below with a focus on new features added to the model, and readers should refer to the original Foodprint documentation [12] for a detailed description of the underlying computations. All model data represent the mean of 2009–2018 to align with contemporary dietary data from the NHANES unless otherwise noted in the model, but users are not limited to these data years. Supplemental documentation can be found elsewhere, which include readme files, analytic code, step-by-step instructions, and supplemental data for 1999–2021 (**S1 Table** and https://doi.org/10.17026/dans-zmh-tzn3). Data in all worksheets represent the US national food system so users interested in modeling other geopolitical scales should use data relevant to their area of interest.

### Input

Users begin by entering information on the population size and trade system (open or closed) of interest. An open trade system is one in which agricultural commodities are imported into and exported out of the study area, which can be useful for evaluating the agricultural resource requirements of food demand in the US, which includes the resources associated with imported food and excludes the resources associated with exported foods. The model accounts for this by incorporating data on the agricultural trade balance of each commodity from the *Trade* worksheet, which is discussed below. A closed system does not account for trade and can be useful for evaluating the capacity for a given food system (at any geographic scale) to feed their populations with food produced within the study area [13]. Data from the *Input* worksheet are used by the *Food requirements* worksheet, which is discussed below.

The *Input* worksheet accepts user-inputted data on the daily intake of 22 food groups: grains; dark green vegetables; red and orange vegetables; dry beans, lentils, and peas; starchy vegetables; other vegetables; fluid milk and yogurt; cheese and other dairy; soy milk; nuts; tofu; beef; pork; chicken; turkey; eggs; aquatic food; plant oils; dairy fats; lard and tallow; and sweeteners. Users have two options for entering these data. First, these data can be entered manually as per capita intakes, which was the original design of the model. Users can acquire data on actual per capita intakes from the US Department of Agriculture’s Loss-Adjusted Food Availability (LAFA) data series, [16] or users can enter theoretical dietary data which can be useful for investigating counterfactual scenarios [12].

Second, users can use a new embedded macro to automate the input of individual-level dietary data from surveys, such as the NHANES or others. Supplemental documentation provides Stata programming code and step-by-step instructions that describe how to prepare the diet input file using NHANES dietary data (https://doi.org/10.17026/dans-zmh-tzn3). This documentation also describes how to use supplemental databases including the USDA Food patterns Equivalents Database [17] and Food and Nutrient Database for Dietary Studies [18] to convert dietary data from mass quantity in NHANES into servings of each food group needed by the model. The macro will iteratively enter each participant’s dietary data into the *Input* worksheet and export the results into a separate file that users can import into their preferred statistical programming package for analysis.

### Dietary recommendations

The calculations in the *Dietary recommendations* worksheet adjust the recommended intake of food groups from the Healthy US Style Dietary Pattern in the 2020–2025 Dietary Guidelines for Americans [19] to the age-sex distribution of the population of interest (the original model used the MyPyramid recommendations). Data from this worksheet are not used by the model’s embedded computations but can be used as a reference in case users want to manually input dietary data that align with recommended intakes into the *Input* worksheet.

### Food preferences

Dietary data are input into the model on the basis of food groups rather than individual foods, so the *Food preferences* worksheet estimates the individual foods that comprise each food group (e.g., dark green leafy vegetables includes spinach and kale) using data on per capita food availability from LAFA [16]. This worksheet allows users to indicate whether a food can be produced within the study area. Foods that cannot be produced within the study area will not be included in the model computations, and the consumption amount for that food will be reapportioned to other foods within that food group in proportion to the per capita consumption amounts of the other foods within that food group. These data are used by the *Food requirements* worksheet.

### Nutrient composition

The *Nutrient composition* worksheet tabulates data on serving sizes (grams per serving) of each food, as well as their content of energy and macronutrients, which were acquired from USDA FoodData Central [20] (the original model used data from USDA Nutrient Database for Standard Reference, which has since been replaced with updated data from FoodData Central). Data on serving sizes are linked to the *Food requirements* worksheet where they are used to convert the servings (ounce equivalents or cup equivalents) of each food consumed to the equivalent mass quantity of agricultural commodities. Data on the nutrient content of each food are linked to the *Output* worksheet where they are used to estimate the energy and macronutrient content of the user-defined diet pattern on a per capita basis, but users interested in estimating individual-level nutrient intakes are recommended to use the nutrient content information from their source data (e.g., NHANES).

### Losses and waste

Data on retail loss, inedible portions, cooking loss, and consumer waste for each food are tabulated in the *Losses and waste* worksheet. These data were acquired from the most recent LAFA [16] (the original model used data from earlier LAFA versions). These data are used by the *Food requirements* worksheet to convert the amount of food consumed to the corresponding amount of agricultural commodities needed.

### Dairy processing

The *Dairy processing conversions* worksheet uses data on the amount of fat and non-fat solids in dairy foods from the USDA Economic Research Service (ERS) [21] and FoodData Central [20] to estimate the total fluid milk requirement in a given diet. Two calculations are used to estimate total fluid milk equivalents using data on fat and non-fat solids, and the larger estimate represents the limiting dairy fraction (i.e., fat solids or non-fat solids) that is used by the *Processing conversions* worksheet. The total fluid milk equivalent (*FM*) based on dairy fat solids (*f*) in a given diet can be expressed as:

$$FM_{fat}=\sum_{i=1}^{N_{D}}\left(Food\;Intake_{i}\times Proportion\;of\;fat\;solids_{i}\right)/0.037,$$

where *Food intake* is the annual per capita consumption amount of each dairy food (*i*), *Proportion of fat solids* is the mass quantity of dairy fat solids in each dairy food as a proportion of its total mass quantity, *N*_*D*_ is the total number of dairy foods included, and the constant 0.037 is the mass quantity of fat solids in fluid milk as a proportion of its total mass quantity.

The total fluid milk equivalent based on dairy non-fat solids (*n*) in a given diet can be expressed as:

$$FM_{non-fat}=\sum_{i=1}^{N_{D}}\left(\;Food\;Intake_{i}\times Proportion\;of\;non-fat\;solids_{i}\right)/0.086,$$

where the constant 0.086 is the mass quantity of non-fat solids in fluid milk as a proportion of its total mass weight.

### Food processing

The *Processing conversions* worksheet estimates coefficients that convert the mass quantity of foods to agricultural commodities, which accounts for losses that occur from processing the raw agricultural commodities into consumer foods. These data were acquired from the USDA ERS [21] and personal communication with experts, as documented in the model and elsewhere [12]. These data are used by the *Food requirements* worksheet to convert the amount of food consumed to the corresponding amount of agricultural commodities needed.

### Fat and oil processing

The *Fat and oil conversions* worksheet estimates the amount of individual fats and oils in processed food products that typically contain multiple types of fats and oils: salad dressing, cooking oil, margarine, shortening, and other edible fats and oils. These data were acquired from the USDA ERS [21, 22]. These data are used by the *Land requirements* worksheet to convert the amount of food consumed to the corresponding amount of agricultural commodities needed.

### Food requirements

The *Food requirements* worksheet uses data on food intake, serving sizes, food preferences, losses and waste, and processing conversions from prior worksheets to convert data on servings of food intake to the equivalent mass quantity of agricultural commodities per year (lb/person/y). This is expressed as:

$$\begin{array}{l} Agricultural\;commodity_{i}=\left(Food\;intake_{i}\times Serving\;size_{i}\right)/(454g/lb)\times Food\;preference_{i}\times \\ \;Loss\;and\;waste_{i}\times Processing\;conversion_{i}\times 365d/y, \end{array}$$

where *Food intake* is the number of servings consumed per capita per day of each food (*i*), *Serving size* (grams/serving) is a coefficient that converts the serving units (cup-equivalents, ounce-equivalents, or teaspoons) of each food to grams, *Food preference* is the adjusted preference of a given food relative to other foods within that food group, *Loss and waste* is a coefficient that adjusts the amount of each food in its as-consumed form to its equivalent gram amount before losses and waste that occur during the retail and consumer stages of the food system, and *Processing conversion* is a coefficient that converts the amount of each food to the equivalent pounds of agricultural commodity.

### Livestock feed requirements

Data in this worksheet report feed conversions that represent the amount of agricultural commodities needed to produce animal products including beef, pork, poultry, dairy, eggs, and farmed aquatic food. These values are used by the *Land requirements* worksheet to estimate the amount of agricultural land needed to produce agricultural commodities.

Feed conversions for beef cattle, dairy cattle used for beef, dairy cattle used for dairy, swine, layers, broilers, and turkeys were acquired from a separate computational simulation model developed by Peters et al. [23] The feed requirements model represents the stocks and flows of each livestock category that includes production animals (e.g., mature productive cows) and support animals (e.g., dry cows and replacement heifers). The nutritional requirements for each life stage were acquired from the National Research Council [24]. A simplified list of the most common feed ingredients included in the model were corn grain, corn silage, soybean meal, hay, haylage, and grazed forage, and their nutrient contents were acquired from DairyOne [25]. Feed rations were balanced to meet energy and crude protein requirements for each livestock category for each life stage. Further details can be found elsewhere [23].

Feed conversions for farmed aquatic food are a new feature to Foodprint 2.0. Aquatic foods from capture fisheries are included in the dietary data entered into the *Input* worksheet but this fraction is not used to estimate agricultural resource requirements because these foods do not rely on agricultural production. Six species that represent 83% of farmed aquatic food consumption in the US [26] were included in the model: carp, catfish, salmon, shrimp, tilapia, and trout. For each species, the share of consumption from farmed versus wild-caught systems was acquired from Kroetz et al., 2020 [27]. The nutritional requirements (*f*: energy and crude protein) for each species (*s*) were estimated as:

$$\begin{array}{l} Nutritional\;requirements_{sf}\;\\ =\;Feed\;conversion\;ratio_{s}\times \mathrm{Pr}ocessing\;efficiency_{s}\times Feed\;ration_{sf}\\ \times Nutrient\;content_{f}, \end{array}$$

where *Feed conversion ratio* is the total weight of feed administered over the lifetime of a species divided by the weight gained [28], *Processing efficiency* is the mass quantity of edible fish per mass quantity of live fish, *Feed ration* is the mass quantity of energy or crude protein needed to produce a given mass quantity of edible fish from MacLeod et al, 2020. [28], and *Nutrient content* is the amount of energy or crude protein per mass quantity of each ingredient from USDA FoodData Central, [20] DairyOne, [25] Feedipedia, [29] and Peters et al., 2014 [23]. Foodprint 2.0 uses corn and soybean meal to satisfy nutritional requirements for each farmed aquatic food species, but users can incorporate additional feed ingredients as needed.

### Trade

Accounting for food trade is a new feature in Foodprint 2.0. Users can indicate whether to model an open or closed food system in the *Input* worksheet, which dictates whether data in the *Trade* worksheet will be incorporated into the model’s calculations. The *Trade* worksheet calculates the trade balance for each food using data on beginning stocks, imports, and exports from the USDA ERS Food Availability data series [30] and USDA Foreign Agricultural Service’s Global Agricultural Trade System [31]. The annual trade balance for each food (*i*) is expressed as:

$$Trade\;balance_{i}=\;Beginning\;stock_{i}+\;Import_{i}+\;Export_{i},$$

where *Beginning stock* is the mass quantity of a given food remaining from the previous year, *Import* is the mass quantity of a given food imported into the study area, and *Export* is the mass quantity of a given food exported from the study area. The trade balance for each food is used by the *Land requirements* worksheet to adjust the amount of agricultural commodities needed for a given diet pattern. A positive trade balance indicates that fewer agricultural commodities are needed for a given diet pattern, and a negative trade balance indicates that additional agricultural commodities are needed.

### Crop and grazing yields

The *Crop and grazing yields* worksheet compiles data on the amount of each crop (and grazing land) produced per acre harvested (in units reported from original sources) and converts them to pounds per acre. These data are used by the *Land requirements* worksheet, and all data were updated from the original version of the model. Data on crop yields were acquired from USDA National Agricultural Statistics Service [32] and unit conversions (from volume or metric units to lbs.) were acquired from USDA ERS [21]. USDA does not report yields from grazing lands (cropland pasture and permanent pasture) so these were estimated using information on the nutrient composition of pasture, total mass quantity of pasture consumed by livestock, and total land area of pasture used for grazing from multiple sources, [33–35] as described elsewhere [12].

### Multiuse crops

Some crops are used to produce multiple end products, such as meal and oil from corn, so taking a simple sum of the land requirements of each end product will lead to an overestimation of total land requirements. The *Multiuse crop adjustment* worksheet prevents this double counting by deriving coefficients (multiuse crop adjustments) that represent the potential land savings attributable to multiuse crops, which are used by the calculations embedded in the *Land requirements* worksheet. This is performed in eight steps which are described below, and all calculations are shown in the worksheet. This worksheet was modified in Foodprint 2.0 to account for the multiuse crops used to feed farmed aquatic food species.

Step 1 of the multiuse crop adjustment estimates the amount of protein from corn gluten used as livestock feed that is co-produced from wet milling whole corn for starch and sweeteners (high fructose corn syrup, glucose, and dextrose) for human consumption. These calculations use data on the amount of corn (as an agricultural commodity) needed to produce a given amount of each type of corn sweetener for human consumption (from the *Food requirements* worksheet), the amount of oil produced from a given amount of corn (from the *Fat and oil conversions* worksheet), the amount of livestock feed produced from a given amount of corn, and the protein content of livestock feed [21, 36].

Step 2 estimates the amount of oil from canola, olives, peanuts, and soybeans produced for human consumption. These calculations use data on the amount of agricultural land needed to produce these crops and their oil content from the *Food requirements* and *Fat and oil conversions* worksheets. Step 3 estimates the amount of protein from soybean meal used as a livestock feed that is co-produced from crushing whole soybeans for oil, which uses data on the amount of each product consumed (from the *Input* worksheet) and the protein content of feeds (from the *Livestock feed* requirements worksheet). This step also estimates the amount of lard and tallow produced from the co-production of each animal-based food for human consumption.

Step 4 computes the multiuse crop adjustment for land used to grow corn by estimating the total amount of corn oil consumed (from the *Food requirements* and *Fat and oil conversions* worksheets) and the amount of corn oil spared (the lowest value between the total amount of corn oil consumed and the total amount of corn oil produced as a co-product of sweetener production from Step 1). Step 5 estimates the remaining amount of lard and tallow needed to satisfy dietary demands (from the *Food requirements* and *Fat and oil conversions* worksheets) after subtracting the total amount of lard and tallow coproduced from the processing of animal-based foods for human consumption (from Steps 1 and 3). Step 6 computes the multiuse crop adjustment for land used to grow oilseeds by accounting for the amount that can be offset by surplus corn oil (from Steps 1, 2, and 4), lard, and tallow (from Step 5). Step 7 estimates the amount of protein for livestock feed coproduced from the processing of soybeans and oilseeds using data from Step 2, the *Fat and oil conversions* worksheet, and USDA ERS [21]. Step 8 computes the multiuse crop adjustment for land used to grow soybeans using data on the coproduction of protein for livestock feed from oilseeds (from Step 7) and corn (from Step 1), and the total protein content of livestock feeds (from Step 3). The data obtained from Steps 4, 6, and 8 (corn and oilseeds spared) is then used to adjust the calculation of land use for these crops in the *Land requirements* worksheet.

### Land requirements

The *Land requirements* worksheet estimates the acres of each type of land (cultivated cropland, cropland pasture, and permanent pasture) needed to meet dietary demands for each food in a given diet. For all foods except fats and oils, this is expressed as:

$$\;Required\;land_{il}=\left[\;Agricultural\;commodity_{i}-\right.\;(Trade\;balance_{i}/Population)]/Yield_{il},$$

For fats and oils, this is expressed as:

$$\begin{array}{l} Required\;land_{il}\;\\ =(Agricultural\;commodity_{i}\\ -Trade\;balance_{i}/Population)\times Fat\;and\;oil\;processing\;conversion_{i}\\ \times Multiuse\;crop\;adjustment_{i}/Yield_{il} \end{array}$$

where *Required land* (acres/person/yr) represents the amount of each type of land (*l*) needed to produce each food (*i*), *Agricultural commodity* is the quantity of each agricultural commodity needed to meet the demand for each food (from the *Food requirements* worksheet) in lbs/yr, *Trade balance* is the total trade balance for each food per year (from the *Trade* worksheet), *Population* is the number of individuals in the study area (from the *Input* worksheet), *Fat and oil processing conversion* is the amount of fats and oils in processed foods, *Multiuse crop adjustment* is an adjustment factor to account for the potential land savings from crops that are used to produce multiple end products, and *Yield* represents the mass quantity of each crop produced per unit of land area for each type of land (lb/acre).

### Amendments

A new feature of the model allows users to estimate the amount of fertilizer nutrients (nitrogen, phosphorus-P_2_O_5_, potash-K_2_O, and sulfur), pesticides (sum of herbicides, insecticides, and fungicides), and irrigation water needed to meet dietary demands. Data on the amount of fertilizer nutrients and pesticides applied to each crop were collected from the USDA Census of Agriculture [37, 38] and the USDA Agricultural Surveys [39, 40]. The Census of Agriculture collects this information from approximately 1.5 million operations with >$1,000 of sales every five years, and data on an additional 500,000 operations is acquired through imputation and calibration. Participation is required by federal law, and data are collected by mail, internet, telephone, and in person [37, 38]. Agricultural Surveys collect data from 65,000–81,000 producers annually, mostly by phone and the remainder are collected by mail and in person [39, 40]. Data on the amount of irrigation water applied to each crop were collected from the USDA Irrigation and Water Management Surveys [41]. All producers who indicated irrigation activity in the Census of Agriculture are contacted by mail every five years, and surveys are submitted by mail, online, telephone, or in person. Approximately 35,000 producers are surveyed and data gaps are filled by statistical imputation and calibration [41].

The *Amendments* worksheet tabulates the mean annual quantity of each amendment applied per unit of land area for each agricultural commodity, adjusted for the proportion of land on which the amendments were applied. These data are used by the *Output* and *Expanded output* worksheets. These values do not include the on-farm resources applied directly to livestock, such as insecticide treatments.

### Land availability

Not all land in farms is suitable for agricultural production, and not all agriculturally productive land is equally suitable for the production of all agricultural commodities. The *Land availability* worksheet performs a series of calculations to estimate the quantity of land suitable for the production of cultivated crops, perennial forages, and grazing, using data acquired from USDA National Agricultural Statistics Service [32] and USDA ERS [34, 42]. The first calculation estimates a coefficient for *Cropping intensity*, which accounts for cases in which multiple crops are produced from the same cropland (otherwise, a simple sum of land area currently dedicated to cultivating each crop would overestimate total cropland). This is expressed as:

$$\begin{array}{l} \;Cropping\;intensity\;\\ =\frac{\;Area\;harvested\;for\;(\;field\;crops\;+\;vegetables\;+\;other\;cultivated\;crops\;)}{\;Total\;area\;in\;cultivated\;crops} \end{array}$$

Total area in cultivated crops excludes the area in farm buildings, roads, rock outcrops, waterways, and wetlands. The area in productive cropland is estimated by:

Productivecropland=Croplandharvested+Croplandusedonlyforpastureandgrazing

and the *Proportion of productive land cultivated* is estimated by:

$$\begin{array}{l} \;Proportion\;of\;productive\;land\;cultivated\;\\ =\;Total\;area\;of\;cultivated\;crops\;harvested/Productive\;cropland, \end{array}$$

where the *Total area of cultivated crops harvested* excludes area on which hay, forage, grass, Christmas trees or short rotation woody crops were harvested. Available cultivated cropland is estimated by:

$$\begin{array}{l} \;Available\;cultivated\;food\;cropland\;\\ =\;Productive\;cropland\times Percentage\;of\;productive\;land\;cultivated\;\\ /Cropping\;intensity\;-\;Non-food\;cropland\;area\; \end{array}$$

where *Non-food cropland area* is the total number of acres harvested of cotton, tobacco, and nursery crops. Available grazing land is estimated by:

$$\begin{array}{l} \;Available\;grazing\;land\;\\ =\;Available\;permanent\;pasture\;+\;Available\;woodland\;used\;for\;grazing,\; \end{array}$$

where *Available permanent pasture* includes land that is used for the production of native (range) and seeded (pasture) forage species that are consumed directly by livestock rather than being cultivated for hay, and *Available woodland used for grazing* represents land that consists mainly of forest that have grass and other forage growth that is used for livestock grazing.

### Grazing land adjustment

Livestock are grazed on land that is generally not suitable for crop production but can also be grazed on cropland when it is available. The calculations in the *Grazing land adjustment* worksheet estimate the amount of cropland that can be used for grazing if all land used for permanent pasture has been used and there is a remaining need for grazing land to meet dietary demands. In the first step, the ratio of available grazing land to available cropland (*RA*) is expressed as:

RA=Availablegrazingland/Availablecropland,

from the *Land availability* worksheet. In the second step, the ratio of required grazing land to required cropland (*RR*) is expressed as:

$$RR=\frac{\sum_{i=1}^{N}\;Required\;grazing\;land_{i}}{\bar{\sum_{i=1}^{N}\left(\;Required\;Cropland_{i}/Cropping\;intensity\;+\;Required\;forage\;land_{i}\right)}}$$

where *Required grazing land* is the quantity of grazing land needed to produce each food (*i*), *Required cropland* is the quantity of all cropland needed to meet dietary demands, *Required forage land* is the quantity of land used for forages needed to meet dietary demands from the *Land requirements* worksheet, and *N* is the total number of foods for which data are available. The third step estimates the ratio of grazing yield from grazing land to grazing yield from cropland (*RY*) as:

$$RY=\;Yield_{g}/Yield_{p},$$

where *Yield* represents the mass quantity of grazed forage harvested by livestock per acre on land used for grazing (*g*) and all cropland (*p*). The fourth step calculates two values that are used by the *Output* and *Expanded output* worksheets. The amount of cropland that is used for grazing is solved by:

$$\begin{array}{l} If\;RR>RA,\;then\;Cropland\;used\;for\;grazing\;\\ =\frac{[\sum_{i=1}^{N}\;Required\;grazing\;land\;-RA\times \sum_{i=1}^{N}(Required\;Cropland_{i}/Cropping\;intensity\;+\;Required\;forage\;land_{i})]}{RA+RY} \end{array}$$

otherwise, *Cropland used for grazing* = 0. and the grazing land offset by cropland grazed is calculated as:

Grazinglandoffset=Croplandusedforgrazing×RY.

### Output

The *Output* worksheet presents summary results of the proportion of agricultural land used by land type (cultivated cropland, total cropland, and grazing land), energy and macronutrient intake, and the amount of agricultural resources (land, fertilizer nutrients, pesticides, and irrigation water) associated with 10 land types (grains, vegetables, fruit, pulses, nuts, feed grains and oilseeds, sweeteners, hay, cropland pasture, and grazing land). Summary results for the use of fertilizer nutrients, pesticides, and irrigation water are a new feature of the model, and these data are also presented visually as pie charts.

The *Output* worksheet also reports the proportion of the population that can be fed a given diet with current resource use efficiencies, which can be used in conjunction with the closed trade system to answer questions about the capacity to localize food production at sub-national scales. It is not recommended that users estimate the proportion of the population fed in an open trade system because the formula used to obtain this value is dependent on a fixed land base in the US which is not relevant when food is imported from outside the US. The number of people that can be fed a given diet is constrained by the land type that is most limited (cultivated cropland, all cropland, and all productive land). The number of people that can be fed (*Population fed*) on each land type is solved by:

$$\begin{array}{l} \;Population\;fed_{cultivated\;cropland}\\ =\;Available\;cultivated\;food\;cropland/\sum_{i=1}^{N_{g}}\;Required\;cultivated\;cropland_{i} \end{array}$$

$$\begin{array}{l} population\;fed_{all\;cropland}\\ =\frac{\;Available\;food\;cropland,\;all\;uses}{\sum_{i=1}^{N_{g}}\left(Required\;cultivated\;cropland_{i}/Cropping\;intensity+\;Required\;perennial\;cropland_{i}\right)}\\ \;+\;Cropland\;used\;for\;grazing \end{array}$$

$$Population\;fed_{all\;productive\;land\;=}$$

$$\begin{array}{l} \frac{\;Available\;food\;cropland,\;all\;uses\;+\;Available\;grazing\;land}{\sum_{i=1}^{N_{g}}\left(\frac{\;Required\;cultivated\;cropland_{i}}{\;Cropping\;intensity}+\;Required\;perennial\;cropland_{i}+\;Required\;grazing\;land_{i}\right)}\\ \;+\;Cropland\;used\;for\;grazing-Grazing\;land\;offset\; \end{array}$$

In each of the above formulas, *N*_*g*_ is the number of food subgroups for which there are data. The lowest of the three *Population fed* values is divided by the population size to estimate the proportion of the population that can be fed a given diet with current resource use efficiencies:

$$\begin{array}{l} Carrying\;capapcity\;\\ =\frac{\;Min(Population\;fed_{cultivated\;cropland},\;Population\;fed_{all\;cropland},\;Population\;fed_{all\;productive\;land})}{Population\;size} \end{array}$$

The total annual quantity of land required to meet dietary demands (*Land use*) is solved by:

$$\begin{array}{l} Land\;use=[(Required\;cultivated\;cropland/Cropping\;intensity)\\ +Required\;forage\;land\times (1\\ +Cropland\;used\;for\;grazing)/Required\;forage\;land\\ +Required\;grazing\;land\times (1-\;Grazing\;land\;offset)/Required\;grazing\;land]\\ \times Population\;size \end{array}$$

The total quantity of each amendment (*b*: fertilizer nutrients, pesticides, and irrigation water) required to meet dietary demands (*Amendment use*) on an annual basis is solved by:

$$Amendment\;use_{b}=\sum_{i=1}^{N_{f}}Land\;use_{i}\times Amendment\;application\;rate_{bi,}$$

where *Land use* is the quantity of land required for each food (*i*) on an annual per capita basis, *Amendment application rate* is quantity used per acre of each amendment (*b*) for each food on an annual basis, and *N*_*f*_ is the number of foods for which data are available.

### Expanded output

The *Expanded output* worksheet is a new feature of the model that presents the amount of agricultural resources (land, fertilizer nutrients, pesticides, and irrigation water) associated with each of 208 foods included in the model. The embedded macro will export all of these data, along with the data from the *Output* worksheet, into a new spreadsheet that users can use to perform analyses in their statistical package of choice.

### Analysis

To demonstrate model performance, individual-level dietary data from NHANES (2009–2018) were read into the model using the macro, which represents national average dietary intake in the US. Data on food intake and other health behaviors are collected continuously from approximately 5,000 non-institutionalized persons per year using a multistage sampling design [43]. Participants complete 1–2 days of dietary recall using the computer-assisted Automated Multiple Pass Method. The present study used data from the first 24-hour recall because this measures per capita intake. Foods reported consumed by respondents were converted to Foodprint categories using the Food and Nutrient Database for Dietary Studies (FNDDS) [44] and Food Patterns Equivalents Database (FPED) [17]. The model exports the results on agricultural resources associated with each individual’s diet pattern into a separate file for statistical analysis. For comparative analysis, dietary data were also averaged for the entire sample (thus representing population-level intake amounts) and hand-entered into the model rather than using the embedded macro to input individual-level intakes.

Diet quality was measured using the Healthy Eating Index (HEI)-2020, which measures compliance with the Dietary Guidelines for Americans 2020–2025 [45]. The HEI-2020 compares the intake of 13 food components (energy adjusted to 1,000 kcal using the density method) against prespecified standards to produce a score for each component that ranges from 0–5 or 0–10. Higher scores are more favorable and intermediate intakes are scored proportionally. The HEI-2020 includes nine components to encourage (total fruit, whole fruit, total vegetables, greens and beans, whole grains, dairy, total protein foods, seafood and plant proteins, and the ratio of unsaturated to saturated fats) and four components to limit (refined grains, sodium, added sugars, and saturated fats). Scores are summed over all components to produce a total score for each individual out of a maximum score of 100 [45].

A Total of 43,079 participants completed a dietary recall, and participants were excluded from the final analyses if they were <1 year of age (n = 2,969), did not provide a reliable dietary recall as deemed by NHANES staff (n = 30), did not consume any food groups used by Foodprint 2.0, (n = 7), and had ≥1 agricultural resource use (land, fertilizer nutrients, or pesticides) that was >3 SD from the mean (n = 689). The final analytic sample included 39,384 participants. All model simulations using Foodprint 2.0 were performed under an open trade system unless specified otherwise. Linear regression models adjusted for energy intake and NHANES survey cycle were used to evaluate the association between diet quality and annual per capita agricultural resource demand, at P<0.05. NHANES survey weights and design variables were used to account for the multi-stage sampling design and to produce nationally representative results for all analyses. The present study was exempted from human studies ethical review by the Institutional Review Board at William & Mary.

## Results

### Agricultural resource use

A total of 238 million hectares (ha; 95%CI: 231–244 million ha) were used for food production annually from 2009–2018 (**Table 1**) to meet the demand for national average diet patterns in the US (**S2 Table**), mostly for beef (77%), followed by dairy (8%). A total of 10,810 million kilograms (kg; 10,678–10,934 million kg) of fertilizer nutrients (N+P_2_O_5_+K_2_O+S) were used annually, mostly for dairy (27%), beef (25%), grains (12%), chicken (7%), and vegetables (5%). A total of 309 million kg (304–314 million kg) of pesticides were used annually, mostly for beef (38%), dairy (18%), and fruit (14%). Annual food production used 71,170 million cubic meters (m^3^; 70,245–72,095 million m^3^) of irrigation water, mostly for beef (31%), dairy (17%), fruit (14%), grains (12%), and vegetables (9%).

**Table 1:** Agricultural resources associated with food production, 2009–2018.

Food category	Land			Fertilizer nutrients^1^			Pesticides^2^			Irrigation water		
	ha × 10^6^		% of total	kg × 10^6^		% of total	kg × 10^6^		% of total	m^3^ × 10^6^		% of total
Total	238	(231–244)	100.00	10,810	(10,687–10,934)	100.00	309	(304–314)	100.00	71,170	(70,245–72,095)	100.00
Grains	10	(9–10)	4.04	1,307	(1,294–1,321)	12.09	12	(12–12)	3.76	8,854	(8,788–8,920)	12.44
Vegetables	1	(1–1)	0.58	552	(539–564)	5.10	15	(15–16)	5.01	6,211	(6,051–6,370)	8.73
Fruit	2	(2–2)	0.77	376	(365–388)	3.48	43	(42–44)	13.92	9,989	(9,686–10,292)	14.04
Dairy	20	(20–20)	8.38	2,951	(2,899–3,002)	27.30	54	(54–55)	17.64	12,282	(12,069–12,496)	17.26
Soymilk	0	(0–0)	0.01	2	(1–2)	0.02	0	(0–0)	0.02	5	(4–6)	0.01
Legumes	1	(1–1)	0.24	92	(87–98)	0.86	2	(2–2)	0.63	1,057	(996–1,118)	1.49
Nuts	1	(1–1)	0.27	97	(93–100)	0.90	11	(11–12)	3.63	2,877	(2,776–2,978)	4.04
Tofu	0	(0–0)	0.02	3	(3–3)	0.03	0	(0–0)	0.03	9	(8–10)	0.01
Beef	184	(177–190)	77.24	2,724	(2,609–2,839)	25.20	117	(112–122)	37.94	21,918	(21,023–22,814)	30.80
Pork	2	(2–2)	0.84	293	(282–303)	2.71	5	(5–5)	1.57	604	(582–626)	0.85
Chicken	5	(5–5)	2.24	768	(744–792)	7.11	13	(12–13)	4.15	1,591	(1,539–1,643)	2.24
Turkey	0	(0–1)	0.21	67	(60–73)	0.62	1	(1–1)	0.38	142	(128–155)	0.20
Eggs	1	(1–1)	0.56	204	(197–210)	1.88	3	(3–3)	1.04	411	(397–425)	0.58
Seafood	1	(1–1)	0.38	128	(116–139)	1.18	2	(2–2)	0.71	268	(243–292)	0.38
Oils	4	(4–5)	1.88	436	(426–445)	4.03	12	(11–12)	3.74	820	(802–838)	1.15
Margarine	0	(0–0)	0.09	15	(15–16)	0.14	0	(0–1)	0.16	45	(44–46)	0.06
Animal fats	4	(4–4)	1.77	493	(472–514)	4.56	11	(10–11)	3.46	1,921	(1,834–2,008)	2.70
Sweeteners	1	(1–1)	0.51	308	(302–314)	2.85	7	(7–7)	2.25	2,181	(2,135–2,226)	3.06

Values in parentheses are 95% CI.

^1^Sum of nitrogen (N), phosphorus (P_2_O_5_), potash (K_2_O), and sulfur (S).

^2^Sum of herbicides, insecticides, and fungicides.

Population-level shifts to higher quality diets, as measured by the HEI-2020 (**Table 2**), were associated with a 0.08 ha (95%CI: 0.09–0.07 ha) decrease in annual per capita land requirements for every 10-point increase in HEI-2020 (P<0.001), driven by a decrease in land associated with beef production (0.08 ha, 0.07–0.09 ha; P<0.001; **S3 Table**). Every 10-point increase in HEI-2020 score was associated with a 0.92 kg (0.7–21.14 kg) decrease in per capita fertilizer nutrient use (P<0.001), mostly driven by a decrease in fertilizer nutrients associated with beef production (1.31 kg, 1.13–1.49 kg; P<0.001) and an increase in fertilizer nutrients associated with fruit production (0.52 kg, 0.51–0.54; P<0.001). Higher HEI-2020 scores were associated with an increase in the use of pesticides (0.01 kg, 0.01–0.02 kg; P = 0.002) and irrigation water (7.11 m^3^, 5.58–8.65 m^3^), mostly attributable to decreased resources associated with beef production (P<0.001 for pesticides and irrigation water) but larger increases in resources associated with fruit production (P<0.001 for pesticides and irrigation water).

**Table 2:** Annual per capita agricultural resources associated with Healthy Eating Index-2020 scores, 2009–2018.

HEI-2020 quintiles (mean)^3^	Land (ha)		Fertilizer nutrients^1^ (kg)		Pesticides^2^ (kg)		Irrigation water (m^3^)	
Quintile 1 (31.07)	0.87	(0.83–0.92)	35.67	(34.78–36.56)	0.94	(0.91–0.98)	211.69	(205.32–218.06)
Quintile 2 (41.33)	0.84	(0.80–0.87)	34.76	(34.10–35.42)	0.96	(0.93–0.99)	216.65	(211.45–221.85)
Quintile 3 (48.75)	0.78	(0.75–0.81)	34.17	(33.60–34.73)	0.97	(0.95–0.99)	221.17	(216.63–225.71)
Quintile 4 (56.53)	0.70	(0.66–0.73)	33.08	(32.42–33.73)	0.97	(0.95–1.00)	225.91	(221.46–230.35)
Quintile 5 (69.60)	0.56	(0.53–0.59)	31.90	(31.38–32.41)	0.99	(0.97–1.01)	237.43	(233.47–241.39)
P-trend	<0.001		<0.001		0.002		<0.001	
Change per 10-point increase in HEI-2020 score	-0.08	(-0.09- -0.07)	-0.92	(-1.14- -0.70)	0.01	(0.01–0.02)	7.11	(5.58–8.65)

Values in parentheses are 95% CI.

HEI-2020, Healthy Eating Index-2020

NHANES, National Health and Nutrition Examination Survey

All values were generated from linear regression models: *Agricultural resource use = β*_*HEI score*_*+β*_*Energy intake*_*+β*_*NHANES survey cycle*_

^1^Sum of nitrogen (N), phosphorus (P_2_O_5_), potash (K_2_O), and sulfur (S).

^2^Sum of herbicides, insecticides, and fungicides.

^3^n = 7,876–7,877 per quintile.

### Foodprint 2.0 vs. Foodprint 1.0

In addition to allowing users to automate the input of individual-level dietary data from surveys such as NHANES using the embedded macro, Foodprint 2.0 retains the functionality of Foodprint 1.0 that allows users to manually input population-level dietary data as mean intakes. Total agricultural land use was 6% (16 million ha) higher when using Foodprint 1.0 compared to Foodprint 2.0, when population-level dietary data were used for both models (**Table 3**). Foodprint 1.0 resulted in 15% (11 million ha) more cropland and 22% (40 million ha) more land for permanent pasture and grazing. The largest percentage increase in cropland was observed for cropland pasture (331% increase), sweeteners (75% increase), vegetables (47% increase), hay (38% increase), pulses (27% increase), and grains (16% increase). The largest percentage decrease in cropland was observed for nuts (17% decrease) and feed grains and oilseeds (3% decrease). A portion of these differences are attributable to new data and calculations in Foodprint 2.0 that account for the land requirements of food trade and farmed aquatic food. But much of these differences can be attributed to updated data on food loss and waste rates in Foodprint 2.0, which are generally lower than the rates used in Foodprint 1.0, particularly for animal-sourced foods that have high land requirements, which thus leads to lower land requirements overall.

**Table 3:** Land use estimated using Foodprint 2.0 and Foodprint 1.0.

Land category	Foodprint 2.0, population-level dietary data, mean (ha × 10^6^)^1^	Foodprint 1.0, population-level dietary data^1^	
		Mean (ha × 10^6^)	% diff.^2^
Total	255	271	6
Cropland	72	83	15
Grains	10	11	16
Vegetables	1	2	47
Fruit	2	2	1
Pulses	0	1	27
Nuts	1	0	-17
Feed grains and oilseeds	30	30	-3
Sweeteners	1	2	75
Hay	23	31	38
Cropland pasture	5	20	331
Permanent pasture and grazing	182	222	22

^1^These results were generated by manually entering the mean dietary intake of all 39,384 participants from NHANES 2009–2018, as presented in S2 Table.

^2^Percent difference from Foodprint 2.0.

### Individual-level vs. population-level dietary data

Compared to the results generated using individual-level dietary data (NHANES individual-level dietary data were fed into the model using the embedded macro), population-level dietary data (NHANES population means) resulted in 7% (17 million ha) more agricultural land used in total, including 23% (35 million ha) more land used for permanent pasture and grazing (**Table 4**). Population-level dietary data resulted in 19% (17 million ha) less cropland, including 69% (10 million ha) less cropland pasture, 17% less land used for vegetables (0.1 million ha), 15% (5 million ha) less land used for feed grains and oilseeds, 13% (0.1 million ha) less land used for pulses, and 11% (0.1 million ha) less land used for nuts. These differences can be attributed to how the model handles extreme dietary intakes, particularly those that require large amounts of land, such as beef and dairy. Population-level dietary data represents the average intake of the entire sample, so extreme intakes are not input individually into the model and therefore do not put as much pressure on the model to find more land to accommodate them. By contrast, individual-level data are input into the model iteratively, and extreme intakes put pressure on the model to find more land to accommodate them. The model does this by using some cropland for grazing, which has higher yield than land used for permanent pasture, thus resulting in less land for permanent pasture and less land overall.

**Table 4:** Land use estimated using individual-level and population-level dietary data.

Land category	Foodprint 2.0, individual-level dietary data, mean, ha × 10^6^ (95% CI)^1^		Foodprint 2.0, population-level dietary data^2^	
			Mean (ha × 10^6^)	% diff.^3^
Total	238	(231–244)	255	7
Cropland	90	(88–91)	72	-19
Grains	10	(9–10)	10	-1
Vegetables	1	(1–1)	1	-17
Fruit	2	(2–2)	2	-6
Pulses	1	(1–1)	0	-13
Nuts	1	(1–1)	1	-11
Feed grains and oilseeds	36	(35–36)	30	-15
Sweeteners	1	(1–1)	1	0
Hay	24	(23–25)	23	-6
Cropland pasture	15	(14–15)	5	-69
Permanent pasture and grazing	148	(142–153)	182	23

^1^These results were generated using the embedded macro in Foodprint 2.0 which iteratively inputs each participant’s dietary data and estimates land use associated with each participant’s diet. The results presented in this table represent the mean land use of all 39,384 participants in NHANES 2009–2018.

^2^These results were generated by manually entering the mean dietary intake of all 39,384 participants from NHANES 2009–2018, as presented in S2 Table.

^3^Percent difference from Foodprint 2.0 using individual-level dietary data.

### Without food trade, farmed aquatic food, and updated loss and waste data

Food trade, aquatic food, and updated loss and waste data are new features of Foodprint 2.0. Removal of food trade (i.e., closed trade system) resulted in 1% (3 million ha) higher total land use, especially for vegetables (44% increase), fruit (14% increase), cropland pasture (13% increase), and pulses (12% increase), and lower land use for nuts (20% decrease) and feed grains and oilseeds (4% decrease; **Table 5**) because less land was needed to satisfy export demands of these foods. Removal of aquatic food resulted in 2% (6 million ha) less total land use, especially for feed grains and oilseeds (10% decrease) but also for permanent pasture and grazing land (2% decrease). Cropland pasture increased by 18%. Removal of updated loss and waste data resulted in 17% (44 million ha) higher total land use, especially for cropland pasture (31% increase), feed grains and oilseeds (26% increase), permanent pasture and grazing (17% increase), and hay (17% increase). Removal of updated loss and waste data resulted in lower land use for fruit (18% decrease) and sweeteners (18% decrease).

**Table 5:** Land use estimated using Foodprint 2.0 without adjustment for food trade, loss and waste, and farmed aquatic food.

	Complete model (ha × 10^6^)	Without food trade		Without aquatic food		Without updated loss and waste data^1^	
Land category		Mean (ha × 10^6^)	% diff.^2^	Mean (ha × 10^6^)	% diff.^2^	Mean (ha × 10^6^)	% diff.^2^
Total	255	258	1	249	-2	299	17
Cropland	72	73	1	71	-2	85	17
Grains	10	10	4	10	0	9	-1
Vegetables	1	2	44	1	0	1	-5
Fruit	2	2	14	2	0	1	-18
Pulses	0	1	12	0	0	0	0
Nuts	1	0	-20	1	0	1	-2
Feed grains and oilseeds	30	29	-4	28	-10	38	26
Sweeteners	1	1	3	1	0	1	-18
Hay	23	23	2	23	0	27	17
Cropland pasture	5	5	13	6	18	6	31
Permanent pasture and grazing	182	185	1	178	-2	214	17

All dietary data are mean per capita intakes from the National Health and Nutrition Examination Survey (2009–2018), as presented in S2 Table. These were manually entered into the model without the embedded macro.

^1^Loss and waste data are from Foodprint 1.0.

^2^Percent difference compared to the complete model that includes food trade, updated loss and waste data, and seafood.

## Discussion

This study introduced Foodprint 2.0, a computational simulation model that estimates the agricultural resource requirements of diet patterns. Using ten years of nationally representative dietary data, this study demonstrated that animal-sourced foods such as beef and dairy accounted for the greatest share of total land use, fertilizer nutrient use, pesticide use, and irrigation water use in the average US diet from 2009–2018, followed by grains and chicken (land use and fertilizer nutrient use), fruit and vegetables (pesticide use), and fruit and grains (irrigation water use). Greater adherence to dietary recommendations was associated with lower demand for land and fertilizer nutrients but higher demand for pesticides and irrigation water.

These findings are consistent with prior research that demonstrated higher agricultural resource requirements of animal-sourced foods, followed by grains, vegetables, and fruit [46–48]. However, eliminating animal-sourced foods is not a requisite for improving the environmental sustainability of diet patterns, [49] and some plant-based diets have mixed impacts on agricultural resource use [46]. Others have shown that shifts toward healthier diets, which include greater quantities of fruits and vegetables, can be associated with greater use of some agricultural resources due to their relatively high resource demand per unit of land area [15].

Foodprint 2.0 has several new features that improve its robustness and usability compared to earlier versions of the model. These include embedded functions to integrate individual-level data from dietary surveys; new data and calculations to account for the land requirements of food trade, farmed aquatic food, and food loss and waste; output data on the use of fertilizer nutrients, pesticides, and irrigation water; output files for over 200 foods as well as summary output charts for each food category; and supplemental files that include annual data for each model parameter over a 20 year period for most foods.

Foodprint 2.0 allows users to input dietary data as individual-level intakes using an automated Visual Basic Analysis program which iteratively inputs each participant’s diet pattern into the model and transcribes the outputs to a new data file for subsequent analysis. This enables variance estimation procedures and statistical testing, which can be useful for comparing the agricultural resource requirements across diet patterns. To facilitate this process, step-by-step instructions for preparing the input data file are provided in the supplemental files along with sample Stata programming code for preparing and analyzing NHANES dietary data. This program uses the USDA Food Patterns Equivalents Database (FPED) to directly estimate the individual-level intake of grains; dark green vegetables; red and orange vegetables; dry beans, lentils, and peas; starchy vegetables; other vegetables; fluid milk and yogurt; cheese and other dairy; soy milk; nuts; plant oils; aquatic food; and sweeteners from NHANES; and the mean intake of these food categories is consistent with those previously published by USDA using FPED [50]. For food categories that cannot be estimated directly using FPED (beef, pork, chicken, turkey, dairy fats, and lard and tallow), the sample code demonstrates how these food categories can be estimated indirectly using the USDA FNDDS, [44] FPED conversion files, [17] and NHANES; [51] and their mean intake is consistent with previously published estimates using similar methods [52–54].

The model also retains the original functionality that allows users to manually input population-level dietary data from any source. Users can analyze the mean intake of food categories from NHANES as described above, or they can use per capita data from the USDA Loss-Adjusted Food Availability (LAFA) data system. However, users should be aware that these dietary data are obtained using different methods (NHANES uses individual-level 24-hour recalls whereas LAFA uses population-level food balance sheets) that can produce different results [55]. Regardless of the source of dietary data, users should be aware that their decision to use individual- or population-level dietary data can also produce different results. The present study demonstrated a modest difference (6%) in total land use depending on whether individual- or population-level dietary data were used, yet the difference was larger for some land use categories. These differences are attributable to how the model handles extreme dietary intakes, particularly those at the right tail of the distribution (i.e., very high intake amounts). Individual-level dietary data are typically skewed right, with many people consuming low amounts of a given food group (bounded by zero) and few people consuming very high amounts (functionally unbounded). This is true even after removing outliers, as was done in the present study. Because each individual’s dietary data is iteratively read into the model, those with extreme intakes put pressure on the model to find land to accommodate them, which was particularly evident for food groups that have high land requirements like beef and dairy, which require large amounts of cropland (hay, grains, and oilseeds) as well as grazing land. The model does not reach the limit of total available cropland so it is able to find more, but the model does reach the limit of available land used for permanent pasture. As a result, the model allows some cropland to be used for grazing, and since grazed cropland has much higher yield than permanent pasture land, this results in much less land needed for permanent pasture and therefore less land overall. By contrast, when using population-level dietary data, these extreme intakes at the right tail of the distribution are averaged with all of the other intakes before being entered into the model rather than being read into the model individually. As a result, the model does not process these extremely high intake amounts individually when using population-level dietary data, so there is not as much pressure exerted on the model to find more land to accommodate these extreme dietary intakes.

Foodprint 2.0 incorporates a new function that allows users to model an open food system that accounts for food trade into and out of the food system, or to model a closed food system that does not allow for trade. The trade function utilizes data on the trade balance for each food category to adjust the land required for each food category, rather than data on crop-specific yields and agricultural resource requirements from each trade partner due to limited data availability. The open food system therefore assumes that imported foods have the same agricultural resource requirements as domestically produced foods; and given that the US food system is among the most productive in the world, [56] this approach may underestimate agricultural resource requirements. A potential area for improvement for future versions of Foodprint is to incorporate country-specific data on amendment application rates for each crop (fertilizer nutrients, pesticides, and irrigation water applied per land area for each crop). This new feature would allow users to estimate the efficiency gains through trade and the share of agricultural resources that are imported versus exported.

Farmed aquatic food accounts for approximately 50% of US aquatic food consumption [27] but the associated resource requirements were not accounted for in Foodprint 1.0. Thus, new data and calculations were added to Foodprint 2.0 to account for the agricultural resources used to produce farmed aquatic food, which led to a 2% increase in total land use. Due to limited data availability on feed rations for many species, six species (carp, catfish, salmon, shrimp, tilapia, and trout) that cumulatively represent 83% of farmed aquatic food consumption in the US [26] were selected to represent total farmed aquatic food in the model. Nutrient requirements (energy and crude protein, with micronutrient requirements assumed to be met with supplements) for each aquatic food species were estimated using data on species-specific feed rations and their nutrient content using data on 17 feed ingredients, and Foodprint 2.0 meets these nutrient requirements with corn and soybean meal. Users can incorporate data on additional nutrient requirements and feed ingredients as these become available.

Data on rates of food loss and waste for each food were acquired from USDA LAFA, which were last updated in 2007 and 2011. Foodprint 1.0 used data from the 2007 update whereas Foodprint 2.0 uses data from the 2011 update. For many foods, particularly animal-sourced foods, these rates decreased from 2007 to 2011, thus estimating that more food was available for consumption; and as a result, this study demonstrated that 17% less total land area was required to meet food demand in the US when data from 2011 were used rather than 2007. This demonstrates that food loss and waste is a key driver of agricultural resource use in the US, and further updates to USDA LAFA may produce different results. This also demonstrates that Foodprint 2.0 can be a useful tool to examine the impact of meeting national food loss and waste reduction targets [57] on agricultural resource use, which warrants further research efforts.

As noted above, due to limited data availability on crop-specific application rates for chemicals and irrigated water from international trade partners, Foodprint 2.0 uses the US national average application rates for imported commodities, which likely underestimates the total use of these amendments. Additionally, data are limited on the chemical application rates for US permanent pasture and grazing land, which were assumed to be zero based on personal communication with experts in top-producing states. However, some farms do apply chemicals to pasture and grazing land; [58, 59] and given that this land accounted for over 70% of total land area in the present study, even small amounts of pesticides and fertilizers applied to this land would result in large amounts of total application. Therefore, the agricultural resource requirements estimated by Foodprint 2.0 should be interpreted as conservative.

Policy agendas at the global and US levels are calling for increased investment in research that evaluates the environmental impact of diets and food systems, [2, 4–6] and computational models like Foodprint 2.0 are essential for this purpose [8]. A salient policy question is whether and how to incorporate sustainability into national dietary recommendations such as the Dietary Guidelines for Americans, which rests on a better understanding of the relationship between diet quality and sustainability. The present study contributes to this discussion by showing that greater adherence to the Dietary Guidelines for Americans (as measured using the HEI-2020) was associated with lower demand for some agricultural resources (land and fertilizer nutrients, as well as a small but statistically significant decrease in pesticides) but not others (irrigation water). Others have shown that higher HEI-2015 scores were associated with lower greenhouse gas emissions for some but not all popular diet patterns, [60] and higher HEI-2015 scores have been associated with higher diet cost [60, 61]. Recent research has also demonstrated that fruits and vegetables, which are pillars of healthy diet patterns, account for as much as 30% of the forced labor risk embedded in the US food supply [62].These findings suggest that a population shift toward healthier diets can lead to sustainability trade-offs, which calls for great care when communicating these nuanced findings to the public to avoid unintended consequences. These findings also highlight the importance of identifying large-scale policy interventions that address all domains of sustainability at the same time rather than piecemeal. Further research is needed to evaluate the association between diet quality and multiple sustainability domains simultaneously, and to measure the sustainability impacts associated with other diet quality indices.

This study has several additional strengths. Foodprint 2.0 fills several gaps that otherwise limit model usability, which include a lack of transparency of data inputs and mathematical formulas, which can prevent their use by researchers and other stakeholders; [7, 10] and a lack of ability to represent diverse geopolitical scales (e.g., community, national, and global) [8, 10]. Foodprint 2.0 is highly modifiable and can be used to evaluate the agricultural resource requirements of diet patterns at the local, regional, and national levels, and can be used to model counterfactual scenarios that align with policy targets. The model is currently parameterized to represent the US national food system so users interested in modeling other geopolitical scales will need to re-parameterize the model accordingly. Supplementary files provide raw and summary data for most parameters on an annual basis from 1999–2018 so users can incorporate the relevant data for their specific research questions. The present study uses data from the USDA Census of Agriculture, which collects detailed farm-level data directly from approximately 1.5 million operations across the US every five years, and imputation and calibration methods account for an additional 500,000 operations [37, 38] This study also uses data from the USDA Agricultural Surveys, which collect annual data from up to 81,000 operators [39, 40]. Given the quality of these data, Foodprint uses them by default but there are other sources of agricultural data that Foodprint can accommodate. For example, others have used region-specific geospatial models to estimate the agricultural land requirements of current and recommended diet patterns, [63] and inputted these estimates into Foodprint to estimate the agricultural capacity to accommodate these dietary shifts [64]. Users also have the option of incorporating individual-level or population-level dietary data, which are supported by sample programming code and step-by-step instructions for use; and to choose between an open or closed food system. Finally, Foodprint 2.0 provides output files for each of 208 food categories which can be aggregated into user-defined food groups for subsequent analysis.

This study also has several limitations. Foodprint 2.0 does not produce variance estimates when population-level dietary data are manually inputted (rather than using the embedded macro to automate the input of individual-level dietary data), but users can use a Monte Carlo simulation for this purpose as demonstrated elsewhere [65]. Data availability is limited for some foods for some parameters, as described above and in the supplemental files, which likely underestimates total agricultural resource requirements, so these should be interpreted as conservative estimates. Further efforts are needed to fill these data gaps. Finally, Foodprint 2.0 uses data on current agricultural activity from the US Census of Agriculture to establish the agricultural land base, but there may be potentially productive land outside of these documented areas [63]. Geographic information systems can be used to identify these lands and their potential productivity, which could add to the agricultural land base in the model.

## Conclusions

Foodprint 2.0 is a computational simulation model that estimates the agricultural resource requirements of diet patterns and can be used to inform sustainable policy agendas at multiple geographic scales. This version includes new data and functions that improve its robustness and usability over previous versions and is highly modifiable so that users can adapt it to address specific research questions. Using ten years of nationally representative dietary data, Foodprint 2.0 demonstrated that animal-sourced foods accounted for the greatest share of agricultural resource use, followed by grains, fruits, and vegetables. Greater adherence to the Dietary Guidelines for Americans was associated with lower use of land and fertilizer nutrients, and greater use of pesticides and irrigation water.

## Supporting information

S1 Table Structure of supporting data files. (XLSX)

S2 Table Mean daily food intake of survey respondents, 2009–2018 (39,384). (XLSX)

S3 Table Annual per capita agricultural resources associated with a 10-point increase in Healthy Eating Index-2020 scores, by food group, 2009–2018. (XLSX)

## Abbreviations

US: United States
USDA: United States Department of Agriculture
ERS: Economic Research Service
FNDDS: Food and Nutrient Database for Dietary Studies
FPED: Food Patterns Equivalents Database
LAFA: Loss Adjusted Food Availability data series
US HHS: United States Department of Health and Human Services
HEI: Healthy Eating Index
NHANES: National Health and Nutrition Examination Survey
